# Incidental detection of microfilaria in cyst fluid of Mucinous cystadenocarcinoma of ovary: A rare case report

**DOI:** 10.1016/j.ijscr.2020.04.051

**Published:** 2020-05-11

**Authors:** Vyshnavi Vasantham, Shakti Kumar Yadav, Namrata Sarin, Sompal Singh, Sonam Kumar Pruthi

**Affiliations:** Department of Pathology, North Delhi Municipal Corporation Medical College and Hindu Rao Hospital, New Delhi, India

**Keywords:** Mucinous cystadenocarcinoma, Microfilaria, Exfoliative cytology

## Abstract

**Introduction:**

Filariasis is a major health problem in certain parts of India. Microfilaria detection on exfoliative and fluid cytology is extremely rare and it is even very rare to find microfilaria coexistent with neoplastic lesions.

**Presentation of case:**

A 45-year-old nulliparous female, who presented with complaints of lower abdominal swelling and pain. Initially, a clinical diagnosis of cystadenoma was made. Ascitic fluid cytology of the patient showed 3-dimensional clusters of cells along with an occasional microfilaria. On histopathology, a diagnosis of Mucinous cystadenocarcinoma of ovary was made.

**Discussion:**

Most common cause of lymphatic filariasis is *W. bancrofti* followed by *Brugia species* and it affects young adults. The adult form of the filaria lodge in lymph vessels, and due to lymphatic blockage in neoplasms they appear in tissue fluid or on surface material. It is the first case report of microfilaria being detected in ovarian cystic fluid cytology in a patient of Mucinous cystadenocarcinoma of ovary.

**Conclusion:**

Filaria is an incidental finding in most of the cases, hence, cytopathologist should be vigilant and careful screening of all the slides should be done, especially in a country like India, where it is highly endemic.

## Highlights

• Microfilaria detection on exfoliative and fluid cytology is extremely rare.
• 45-year-old nulliparous female presented with complaints of lower abdominal swelling and pain.
• Ascitic fluid cytology showed 3-dimensional clusters of cells along with microfilaria.
• On histopathology, a diagnosis of Mucinous cystadenocarcinoma of ovary was made.

## 1 Introduction

Filariasis, is a major health problem in India especially in eastern region and its coastal areas. India contributes around 40% of the total global disease burden and about 50% of people are at risk of infection [1,2]. Lymphatic filariasis is mainly caused by nematodes such as Wuchereria bancrofti (W. bancrofti), Brugia malayi and Brugia timori. W. bancrofti is the most common filarial infection globally [3]. It is highly unusual to detect microfilaria on fluid cytology and in malignant effusions. Microfilaria have been previously reported in sites such as breast, thyroid, epididymis and lymph nodes [4,5], bone marrow, gynecological smears, soft tissue swellings, liver, and body fluids [6,7].

Also, there are no reported cases of microfilaria in ovarian cyst fluid in patients with co-existing ovarian neoplasms. Adult filarial worm frequently lodge in lymph nodes and in lymphatic plexus of the male genitalia. However, filariasis of the female genital tract is extremely rare [8].

Here, we present a very rare case of mucinous cystadenocarcinoma of ovary in which ovarian cyst fluid cytology of the same patient demonstrated microfilaria. This case is reported in line with the SCARE criteria [9].

## 2 Case report

A 45-year-old nulliparous female presented with lower abdominal pain and swelling to Gynaecology OPD. On per vaginal examination, a mass was felt in right fornix. Rest of the general physical and systemic examination were within normal limits.

CT-scan of abdomen showed an heterogenous right adnexal mass measuring 20 × 20 cm along with mild ascites, hepatomegaly and hepatic hemangioma.

Based on the examination and radiological findings, a clinical diagnosis of ovarian cystadenoma was made. Right ovarian cystectomy was performed and was sent to histopathology department.

Also, received were peritoneal fluid and intraoperative ovarian cyst fluid for cytological evaluation.

### 2.1 Cytological examination

**Fig. 1:**
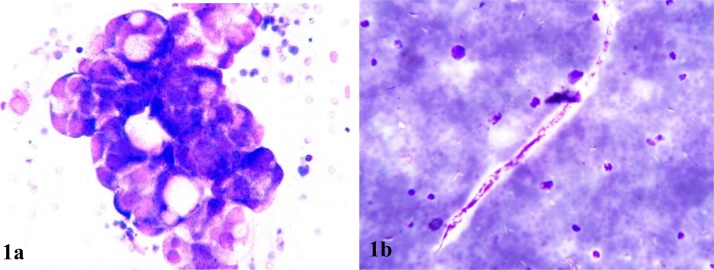
Cells arranged in 3D clusters (A; MGG,40X) Microfilaria (B; MGG,40X).

Cyst fluid was haemorrhagic on gross examination and on microscopy three dimensional clusters of cells with smooth outline were visualized. These cells had large hyperchromatic nucleus with scant cytoplasm. An occasional microfilaria was identified in the haemorrhagic background [Fig. 1].

### 2.2 Histopathological examination

#### 2.2.1 Gross examination

**Fig. 2:**
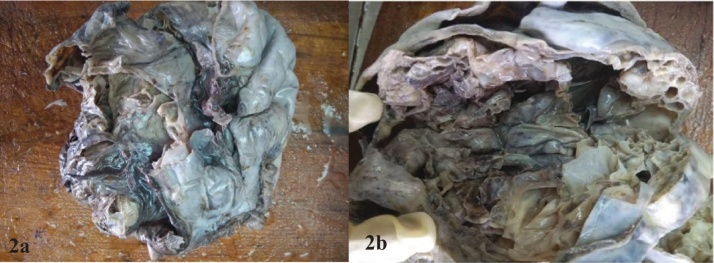
Grey white to grey brown solid and cystic mass with multiloculated cystic cavities being filled with mucoid material and serous fluid.

Outer surface of the ovarian mass was glistening grey white with solid, cystic and congested areas. Cut surface showed multiloculated cysts of varying sizes which were filled with mucoid material and serous fluid [Fig. 2].

**Fig. 3:**
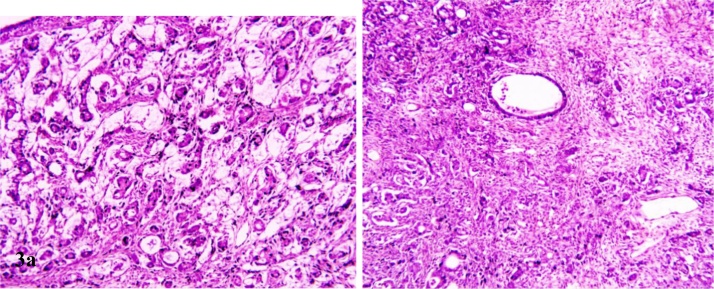
Clusters, nests and single cell pattern of tumor cells with intervening septa (H&E,40X) brown areas. Cystic.

**Fig. 4:**
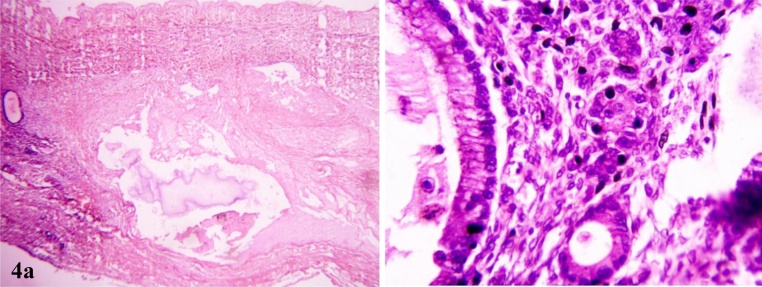
Necrosis (H&E,10X) and lining of mucinous cyst demonstrating picket fencing (H&E,40X).

**Fig. 5:**
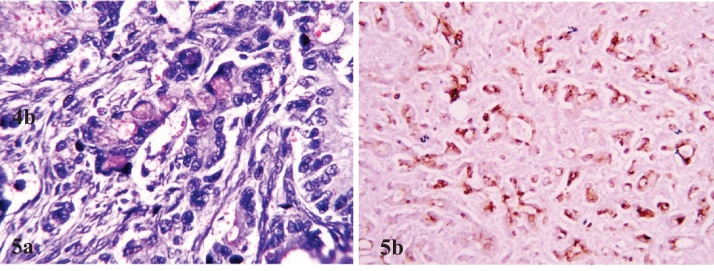
Tumor cells demonstrate mucicarmine positivity (MUC,40X) and are positive for CK-7 (40X).

Multiple sections studied from ovarian mass showed a tumor comprising of tumor cells arranged in clusters, glandular and in singly scattered pattern. Tumor cells have round to oval vesicular nucleus with moderate amount of eosinophilic cytoplasm and showed infiltration into ovarian stroma [Figs. 3 & 4]. Mucicarmine stain highlighted intracellular and extracellular mucin in tumor cells [Fig. 5a]. Immunohistochemical panel of CK-7, CK-20, p53, Ki-67 was applied. Tumor cells demonstrated strong CK-7 [Fig. 5b] and weak CK-20 positivity. Ki67 was markedly high. However, p53 was negative. On the basis of fluid cytology, gross, histopathological and immunohistochemical findings a final diagnosis of “Mucinous cystadenocarcinoma of ovary with microfilaria in ovarian cyst fluid” was made.

### 2.3 Discussion

Filariasis is a major public health issue in south east Asia including Indian subcontinent and tropical region. In India, the most common cause of lymphatic filariasis is *W. bancrofti* followed by *Brugia species*. It affects young adults. It is transmitted through the bite of Culex mosquito [10]. It is endemic in Uttar Pradesh, Bihar, Orissa, Jharkhand, Gujarat, Andhra Pradesh, Tamil Nadu and Kerala [11].

Two hosts are present in the life cycle of nematodes, female Culex mosquito as intermediate host and humans as definitive host. Microfilariae are the infective agent. Larvae forms from the mosquito enter the lymphatics of humans and develop into adult worms. They reside in lymphatics and release microfilaria into the peripheral blood to be taken up by another mosquito [12].

Microfilaria of *W. bancrofti* has a sheath longer than the larval body and nuclei are of equal size, countable and the tail tip is free of nuclei. *Brugia malayi* shows secondary kinks with its larval form showing coalescing uncountable nuclei, extending into the tail tip. Microfilariae on MGG slides appears shorter and stouter, and, on Papanicolaou-stained slides they appear longer and slenderer. Adult worm of *W. bancrofti* resides in lymphatic vessels, and microfilariae is found in peripheral blood (Table 1).

**Table 1:** Morphological features of species causing filariasis [13].

Species	Sheath Appearance	Tail nuclei
Wuchereria Bancrofti	Present; Sweeping curves	Do not extend to tip of tail, tail tapers to delicate point
Brugia Malayi	Present; Stiff with secondary kinks	Subterminal & terminal, tail constricted at 2 terminal nuclei
Brugia Timori	Present; Tapering gradually	Subterminal and terminal
Loa Loa	Present	Continuous to tip of tail
Mansonella Persitans	Absent	Continuous to tip of tail
Mansonella Ozzardi	Absent	Do not extend to tip of tail

The adult form of the filaria lodge in lymph vessels, and due to lymphatic blockage in neoplasms they appear in tissue fluid or on surface material. Microfilaria can also present as dermatitis and skin nodules. Microfilaria has been reported as an incidental finding in neoplastic lesions [14].

Pantola et al. [15] reported seven cases where microfilaria was found in cytological smears at rare sites such as thyroid, parotid, breast, gall bladder, lung, bone, neck secondaries with primary from larynx. Gupta et al. [16] reported five cases of microfilaria as an incidental finding in cavernous hemangiomas, transitional cell carcinoma, Non-Hodgkin’s lymphoma, follicular carcinoma of thyroid and germ cell tumor of testis.

Sane and Patel reported a case where adult filarial worm was identified in cystic teratoma of ovary [17]. Mali BN et al. [18] reported microfilaria as an incidental finding in cervicovaginal smears. Sethi et al. [19] and Goel et al. [20] reported a case of microfilaria as an incidental finding in ovary.

Shubham S et al. [20,21] reported two cases of microfilaria in kidney biopsy. Mohan N et al. [22] reported a case of infiltrating ductal carcinoma of breast with coexistent microfilaria.

Few publications are available reporting coexistence of microfilaria and malignant neoplasms/malignant effusion on cytosmear. Concentration of parasites in neoplasms occurs due to their rich vascular supply [16].

To the best of our knowledge, it is the first case report of microfilaria being detected in ovarian cystic fluid cytology in a patient of Mucinous cystadenocarcinoma of ovary.

### 2.4 Conclusion

Filaria is an incidental finding in most of the cases, hence, cytopathologist should be vigilant and careful screening of all the slides should be done, especially in a country like India, where it is highly endemic. Also, the presence of unexplained acute and chronic inflammatory cell infiltrate in the exfoliative cytology specimen of neoplastic effusions requires a thorough search for helminths.

## Declaration of Competing Interest

None declared.

## Sources of funding

No funding has been received.

## Ethical approval

Exempted as ethical approval not required.

## Consent

Informed written consent was taken from the patient for the publication of this manuscript and any images associated with it.

## Author contribution

Study concept or design - Dr Sonam.

Data collection - Dr Shakti.

Data analysis or interpretation - Dr Sompal and Dr Namrata.

Writing the paper - Dr Vyshnavi.

## Guarantor

Dr Sonam Kumar Pruthi.

## Registration of research studies

NA.

## Provenance and peer review

Not commissioned, externally peer-reviewed.
